# The Reliability and Validity of Short Form-12 Health Survey Version 2 for Chinese Older Adults

**Published:** 2019-06

**Authors:** Shu-Wen SU, Dong WANG

**Affiliations:** School of Health Services Management, Southern Medical University, 1023 Shatai Road, Guangzhou 510515, China

**Keywords:** Health-related quality of life, Older adults, Reliability, SF-12v2, Validity

## Abstract

**Background:**

We assessed the information regarding the psychometric properties of the Short Form-12 Health Survey Version 2 (SF-12v2) among older adults in China

**Methods:**

A cross-sectional study was conducted on a stratified representative sample of older adults (≥60 years) residing in community and nursing home settings in 2017–18. Reliability was estimated using the internal consistency method. Validity was assessed using convergent and discriminant validity checks, factor analyses (including both exploratory and confirmatory factor analyses [EFA and CFA]), and “known groups” construct validity.

**Results:**

The final sample comprised 1000 older adults (451 community-dwelling and 549 institutional). Cronbach’s α was 0.81 for the Physical Component Summary (PCS) and 0.83 for the Mental Component Summary (MCS), showing satisfactory internal consistency for both. Most items were strongly correlated with their represented component (Spearman’s correlation coefficient: 0.62–0.87), although the correlation of SF items with PCS was a bit stronger than that with MCS. A two-factor structure (physical and mental health) indicated by EFA jointly accounted for 68.50% of the variance and presented adequate goodness-of-fit indices (GFI=0.98, AGFI=0.92, RMSEA=0.08, 90% Cl RMSEA=0.06 to 0.11, NFI=0.98, and CFI=0.98) in CFA. Known-groups comparison showed that SF-12v2 summary scores did well in differentiating subgroups of older adults by age, marital status, and self-reported health problems (*P*≤0.05).

**Conclusion:**

SF-12v2 is a reliable and valid health-related quality of life instrument for Chinese older adults that works equally well with older adults under institutional care and community-based home care models.

## Introduction

**Fig. 1:**
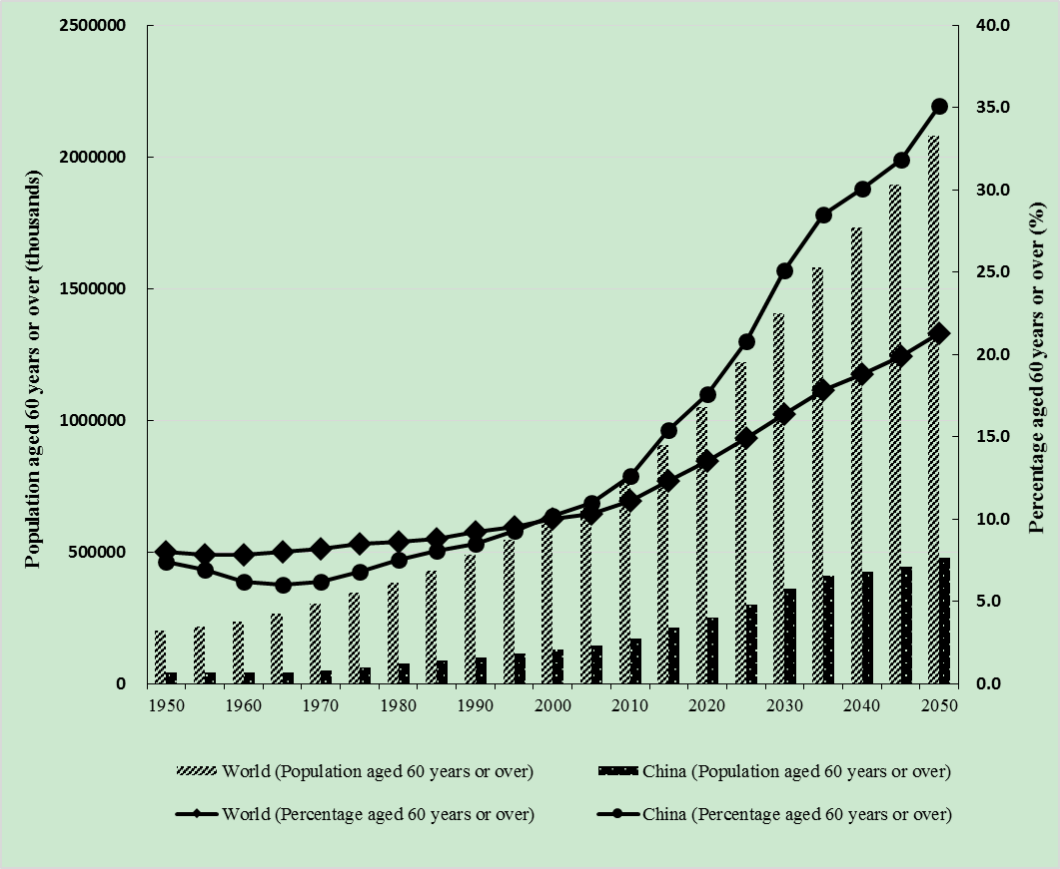
Absolute numbers and proportions of older adults in the world and China. The data for China do not include Hong Kong, Macao, or Taiwan. Data source: United Nations (2017). World Population Prospects: The 2017 Revision

According to the United Nations Population Division, the proportion and absolute number of older adults are increasing dramatically around the world (Fig. 1) (1, 2). China is one of the fastest-aging countries (3). Nearly one-quarter of people aged 60 years or over in 2017 lived in China, which also has the largest number of persons aged 80 or over (1). As the population ages rapidly, growing demand for healthcare is inevitable (4). Health-related quality of life (HRQoL) can be used to measure the effects of healthcare interventions and provide quality-improvement-facilitating outcomes for policy-makers and healthcare providers (5).

The Short Form-12 Health Survey Version 2 (SF-12v2), an abridged practical version of the 36-item Short-Form Health Survey Version 2 (SF-36V2) is widely used in measuring HRQoL (6). SF-12v2’s brevity is an advantage in assessing older adults’ HRQoL in large-scale studies, resulting in less burden for respondents and researchers (7). SF-12v2 is a reliable, valid measure in a variety of population groups (6, 8–12), but information regarding its psychometric properties among older adults in China is still lacking. Two recent studies in Hong Kong have demonstrated validity and reliability for the general population and adolescents there (6, 13). However, no study has verified the instrument’s usability with older adults in China proper.

We used a large sample of Chinese older adults to confirm the psychometric performance (reliability and validity) of SF-12v2 in this subpopulation, so that it can be confidently applied among them to promote healthy and active aging.

## Materials and Methods

### Sample and data collection

A cross-sectional study was conducted from June 2017 to January 2018 among a sample (≥60 years) residing in community and nursing home settings in Guangzhou, the capital of Guangdong Province. According to the WHO and the United Nations (14), people over 60 years of age in developing countries are defined as older adults. The elderly population of Guangzhou (17.3% in 2015, or 1,475,300 older adults) is slightly above the national average; they constituted the survey population (15). Due to economic and cultural similarities, our research results may be extended to other Chinese people in economically developed regions of China (e.g., eastern coastal cities). Dementia patients, psychosis patients, and older adults with cognitive or communicative impairments were excluded.

In community settings, this study used a three-stage sampling method. In the first stage, six districts were selected randomly according to elderly population size to provide a representative sample. In the second, one to three communities were randomly selected by systematic sampling. In the third stage, qualified participants were selected through simple random sampling in each community. In nursing home settings, a two-stage sampling method was applied. First, two nursing homes large enough to provide a representative sample (nursing beds≥1000) were selected randomly. Next, eligible participants were randomly selected from the accommodation registration record in each nursing home. To each of community settings and nursing home settings, 800 questionnaires were distributed. One thousand and fourteen voluntary participants, out of 1600 initially approached (total response rate 63.4%; response rate for community settings 57.0%; response rate for nursing home settings 69.8%), agreed to be surveyed face to face by trained investigators.

Informed consent was obtained from all participants.

### Questionnaire and scoring

The simplified Chinese SF-12v2, authorized by OptumInsight Life Sciences, Inc., was applied in our study. It contains 12 items evaluating eight subscales pertaining to HRQoL, and is a shorter version of the SF-36V2 Health Survey (which is a revised version of the original SF-36 Health Survey). The eight subscale scores (physical functioning [PF], role-physical [RP], bodily pain [BP], general health [GH], vitality [VT], social functioning [SF], role-emotional [RE], and mental health [MH]) range from 0 to 100 (higher scores indicating better health status). Besides scores on the 8 subscales, SF-12v2 gives estimates of summary scores for physical (PCS-12) and mental components (MCS-12), ranging from 0 to 100 (higher scores indicating better physical and mental health), with mean of 50 and SD of 10, calculated by QualityMetric Health Outcomes Scoring Software 5.1 using the US-norm-based scoring algorithm.

General information was investigated in a self-developed questionnaire, including aged care model, age, gender, education level, marital status, chronic diseases, etc. Self-report questions about chronic diseases and health problems were asked by trained investigators recruited from medical university and thus having good knowledge of chronic diseases. Participants were asked “Has a doctor ever told you that you had chronic diseases (for example, hypertension, diabetes, heart problem, osteoarthritis, eye problem, ear problem or others)?” Among the above questions, eye problems included age-related maculopathy, glaucoma, cataract, etc., while ear problems included otitis media, difficulty hearing, etc.

### Quality control

The questionnaire was administered face to face; answers were checked onsite. Respondents were asked to correct or complete any double or missing answers. To ensure data quality, remaining invalid questionnaires, defined as those including missing data or double answers, were excluded from the analysis.

### Statistical analysis

Descriptive analysis for continuous variables was performed using means and standard deviations, while categorical variables were reported using frequencies and percentages. Floor and ceiling effects of PCS-12 and MCS-12 were determined by percentages of sample participants with lowest and highest possible scores.

For the total sample and subgroups under different aged care models, reliability was estimated using the internal consistency method; Cronbach’s α coefficient equal to or greater than 0.70 was considered satisfactory (11).

Validity was assessed using convergent and discriminant validity checks, factor analyses (exploratory factor analysis [EFA] and confirmatory factor analysis [CFA]), and “known groups” construct validity.

In terms of convergent validity, all hypothesized item–component correlations, corrected for over-lap, should be 0.40 or above (16). In terms of discriminant validity, hypothesized item–component correlations should be significantly higher than the alternative item–component correlations (10). Spearman’s correlation coefficient (ϱ) was used to calculate the correlations.

Exploratory factor analysis and confirmatory factor analysis were used to extract the factor structure of SF-12v2. For the total sample and the subgroups under different aged care models, EFA was performed using principal components analysis with varimax rotation. For legitimacy of the analysis, we confirmed that the KMO index was >0.70, and Bartlett’s sphericity test provided a significant result. It was assumed that two principal components would be obtained with eigenvalues greater than 1. For the total sample and subgroups under different aged care models, CFA was performed using a two-factor model (PCS and MCS), which was the theoretical structure of SF-12v2 (10). Acceptable goodness-of-fit values included goodness-of-fit index (GFI), adjusted goodness-of-fit index (AGFI), normed fit index (NFI), and comparative fit index (CFI) more than 0.9 and root–mean–square error of approximation (RMSEA) values less than 0.08 (17).

“Known groups” construct validity was assessed by testing hypothesized relationships between subgroups of the study sample and SF-12v2 component scores. It was expected that older participants, widowed or divorced persons, and those with one or more chronic diseases would report poorer health (7, 10, 18, 19). The t-test was used for comparison.

Statistical analysis was carried out using SPSS version 20.0 (Chicago, IL, USA) and AMOS version 22. The datasets used are available from the corresponding author on reasonable request.

## Results

In total, 1600 older adults from community settings and nursing home settings were approached. Of these, 1014 agreed to take part in the investigation, for a response rate of 63.4%. Among them, 1000 valid questionnaires were included in the analysis, while 14 invalid questionnaires in which missing or double answers were found were deleted. These 1000 participants (66.8% women) were aged from 60 to 108 years (Mean=78.34; SD=9.38); 45.1% came from community settings and 54.9% from nursing home settings. The overall education level of the older adults was low: 14.9% were illiterate (had less than elementary school education), 26.7% had elementary school education, 18.6% middle school, 18.2% high school, and 21.6% an associate degree or higher. The data were 100% complete in the valid questionnaires, benefiting from the face-to-face administration and the strict definition of invalid questionnaires.

Table 1 presents descriptive statistics for the Chinese SF-12v2 scales.

**Table 1:** Distribution and reliability of the Chinese SF-12v2 Health Survey among older adults in Guangzhou (N=1000)

***Scales***	***Mean***	***SD***	***Minimum (% floor)***	***Maximum (% ceiling)***	***Cronbach’s alpha†***		
					***Total (N=1000)***	***Institutional care (N=549)***	***Community-based home care (N=451)***
PF	48.6	39.57	0.00 (31.40)	100.00(26.90)	0.92	0.94	0.88
RP	51.13	30.74	0.00 (12.80)	100.00 (13.10)	0.96	0.97	0.92
BP	62.85	26.96	0.00 (3.70)	100.00 (21.20)	1.00	1.00	1.00
GH	36.65	25.51	0.00 (13.80)	100.00 (1.10)	1.00	1.00	1.00
VT	58.6	27.12	0.00 (4.70)	100.00 (14.30)	1.00	1.00	1.00
SF	59.88	32.29	0.00 (10.80)	100.00 (23.80)	1.00	1.00	1.00
RE	64.45	26.14	0.00 (3.40)	100.00 (18.30)	0.93	0.96	0.87
MH	70.06	20.89	0.00 (0.50)	100.00 (13.00)	0.73	0.80	0.60
PCS	39.92	10.33	16.95(0.00)	67.02(0.00)	0.81	0.79	0.81
MCS	49.08	9.85	12.50(0.00)	72.80(0.00)	0.83	0.84	0.79

† Cronbach’s alpha for PCS was calculated from PF, RP, BP and GH, and for MCS was calculated from VT, SF, RE and MH. SF-12v2: Short Form-12 Health Survey Version 2; MCS: Mental Component Summary; PCS: Physical Component Summary; PF: Physical Functioning; RP: Role Physical; BP: Bodily Pain; GH: General Health; VT: Vitality; SF: Social Functioning; RE: Role Emotional; MH: Mental Health.

Reliability estimates for all subscales exceeded 0.70, indicating satisfactory results (i.e., in the total sample group, Cronbach’s αs for PF, RP, GH, VT, SF, RE, and MH were 0.92, 0.96, 1.00, 1.00, 1.00, 1.00, 0.93, and 0.73, respectively). In the total sample group, internal consistency reliability was 0.81 and 0.83 in PCS and MCS domains, indicating high internal consistency; no floor or ceiling effects were observed in either PCS-12 or MCS-12, implying that SF-12v2 items captured the full range of health states among older adults. For the institutional care group, Cronbach’s αs for PCS and MCS were 0.79 and 0.84, and for the community-based home care group, 0.81 and 0.79. The Chinese SF-12v2 thus showed high internal consistency in these two subgroups.

As shown in Table 2, all correlations between items and hypothesized scale ranged from 0.87–1.00, significantly higher than the alternative item–scale correlations. What is more, correlation analysis showed that items in PF, RP, BP, GH, and SF subscales correlated more strongly with the PCS-12 score than MCS-12 score, with correlations ranging from 0.62–0.87, while items in VT, RE, and MH correlated higher with MCS-12, with correlations ranging from 0.63–0.82.

**Table 2:** Item–scale correlation matrix for the eight SF-12v2 scales and summary measures†

	***PF***	***RP***	***BP***	***GH***	***SF***	***RE***	***VT***	***MH***	***PCS***	***MCS***
PF										
PF1	0.97	0.78	0.45	0.45	0.67	0.47	0.58	0.31	0.87	0.30
PF2	0.96	0.75	0.43	0.44	0.62	0.44	0.52	0.26	0.86	0.24
RP										
RP1	0.77	0.98	0.46	0.54	0.73	0.61	0.61	0.38	0.82	0.46
RP2	0.78	0.98	0.46	0.54	0.72	0.58	0.60	0.35	0.83	0.43
BP	0.45	0.47	1.00	0.41	0.43	0.39	0.43	0.32	0.65	0.29
GH	0.46	0.55	0.41	1.00	0.50	0.47	0.55	0.40	0.62	0.46
SF	0.67	0.74	0.43	0.50	1.00	0.56	0.65	0.45	0.66	0.62
RE										
RE1	0.47	0.60	0.37	0.45	0.56	0.96	0.45	0.61	0.38	0.78
RE2	0.45	0.57	0.37	0.46	0.53	0.97	0.44	0.60	0.36	0.78
VT	0.57	0.62	0.43	0.55	0.65	0.46	1.00	0.49	0.60	0.63
MH										
MH1	0.23	0.28	0.27	0.32	0.32	0.48	0.45	0.87	0.12	0.71
MH2	0.33	0.41	0.32	0.41	0.51	0.66	0.46	0.89	0.22	0.82

† Figures are Spearman’s correlation coefficient (rho).

Higher correlations of each item with a SF-12v2 domain or component are indicated in bold.

All correlations were significant at P≤0.01. Correlation values of 0.40 or above were considered satisfactory.

SF-12v2: Short Form-12 Health Survey Version 2; MCS: Mental Component Summary; PCS: Physical Component Summary; PF: Physical Functioning; RP: Role Physical; BP: Bodily Pain; GH: General Health; VT: Vitality; SF: Social Functioning; RE: Role Emotional; MH: Mental Health

**Fig. 2:**
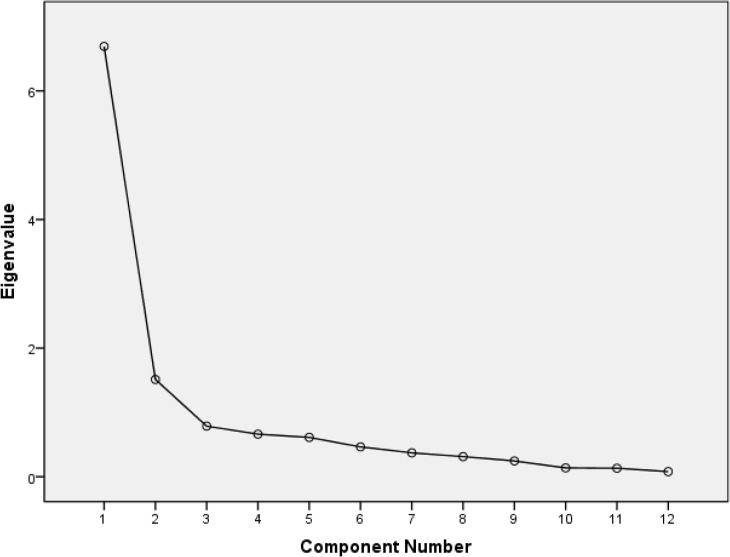
Scree plot of eigenvalues of individual factors of items from the SF-12v2 among Chinese older adults (N=1000)

In the total sample group, the distribution of items on SF-12v2 allowed the use of EFA (KMO=0.90; Bartlett’s *P*<0.001). The two-factor conceptual structure of SF-12v2 items in the Chinese elderly population was confirmed by the scree plot (Fig. 2) and principal components analysis (Table 3).

**Table 3:** Factor structure of the SF-12v2 derived from principal components analysis†

***Variable***	***Total (N=1000)***		***Institutional care (N=549)***		***Community-based home care (N=451)***	
	***Factor 1***	***Factor 2***	***Factor 1***	***Factor 2***	***Factor 1***	***Factor 2***
Physical functioning (PF)						
PF1	0.89†	0.14	0.90†	0.15	0.85†	0.10
PF2	0.88†	0.09	0.88†	0.10	0.85†	0.04
Role physical (RP)						
RP1	0.87†	0.30	0.88†	0.32	0.82†	0.28
RP2	0.87†	0.27	0.88†	0.31	0.84†	0.21
Bodily pain (BP)						
BP1	0.52†	0.27	0.46†	0.29	0.67†	0.23
General health (GH)						
GH1	0.53†	0.39	0.49†	0.48	0.54†	0.26
Social functioning (SF)						
SF1	0.75†	0.38	0.73†	0.43	0.69†	0.34
Role emotional (RE)						
RE1	0.42	0.77†	0.33	0.83†	0.50	0.69†
RE2	0.39	0.78†	0.34	0.83†	0.43	0.70†
Vitality (VT)						
VT1	0.63†	0.43	0.63	0.43	0.58†	0.43
Mental health (MH)						
MH1	0.07	0.78†	0.14	0.79†	−0.01	0.75†
MH2	0.21	0.84†	0.20	0.87†	0.18	0.78†
Eigenvalues	6.69	1.51	6.84	1.60	6.09	1.43
Variance explained (%)	41.40	26.97	39.86	30.48	40.18	22.50

Eigenvalues for the two factors (physical and mental health) that explained most of the variance observed were 6.69 and 1.51 respectively; this two-factor structure jointly accounted for 68.37% of variance.

Principal components analysis, after varimax rotation, showed that items assessing PF, RP, BP, GH, and SF domains loaded higher on the physical component, whereas items assessing MH loaded higher on the mental component. VT and RE items loaded on both components. The two-factor conceptual structure was also confirmed in the subpopulations under different aged care models, which showed similar results to the total sample group in principal components analysis (Table 3).

**Fig. 3:**
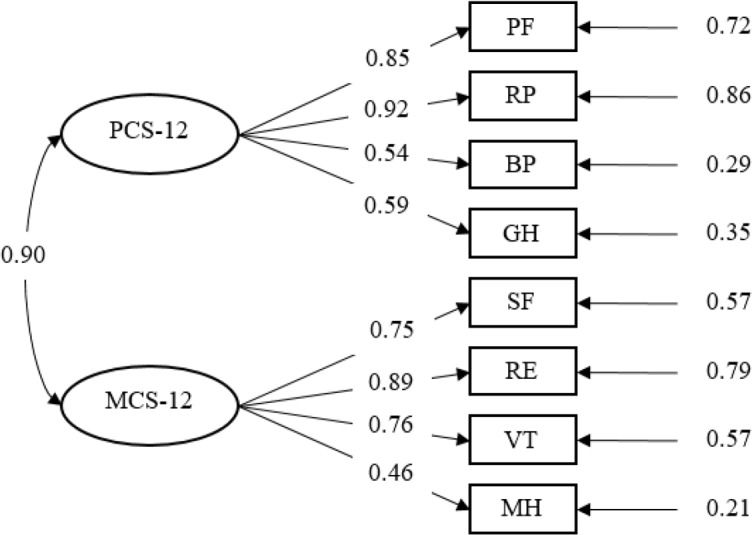
A two-factor model for the SF-12v2 obtained from confirmatory factor analysis (N=1000). Standardized coefficients obtained from the two-factor model in confirmatory factor analysis are presented; all of them are significant at *P*≤0.001

In the total sample group, the results for confirmatory factor analysis of the two-factor model are shown in Fig. 3. The proposed two-factor model, which included PCS-12 and MCS-12, produced adequate goodness-of-fit indices (GFI=1.00, AGFI=0.97, RMSEA=0.05, 90% Cl RMSEA=0.03 to 0.08, NFI=1.00, and CFI=1.00) in confirmatory factor analysis.

The hypothesized model thus conformed well to the empirical data. In the institutional care group, the proposed two-factor model, which included PCS-12 and MCS-12, produced adequate goodness-of-fit indices (GFI=0.99, AGFI=0.94, RMSEA=0.07, 90% Cl RMSEA=0.04 to 0.10, NFI=0.99, and CFI=0.99) in confirmatory factor analysis. For the community-based home care group, this model produced adequate goodness-of-fit indices (GFI=0.99, AGFI=0.95, RMSEA=0.06, 90% Cl RMSEA=0.02 to 0.09, NFI=0.99, and CFI=0.99) in confirmatory factor analysis. It demonstrated that the SF-12v2 worked equally well with subpopulations under these two aged care models.

Significant differences were observed between PCS-12 summary scores by age group (Table 4): the oldest people (age≥80 years old) scored lower than the “younger older” adults (age=60–79 years old; *P*≤0.001). Furthermore, respondents who were widowed/divorced/single/separated demonstrated significantly lower scores than married older adults on both SF-12v2 component scores (*P*≤0.01). In addition, compared with respondents reporting specific health problems, those without such problems exhibited significantly higher mean PCS-12 and MCS-12 scores (Table 4). As seen in Table 4, each specific health problem was significantly associated with reduction in at least one summary score, indicating that SF-12v2 is responsive to the presence of specific health problems among respondents.

**Table 4:** Mean (SD) SF-12v2 summary scores for the elderly population by age, marital status, and self-reported health problems

**Variable (condition)**	***SF-12v2 summary scores***		
	***N (%)***	***PCS-12***	***MCS-12***
**Age groups(yr)**			
60–79	459 (45.90)	42.85 (10.02)	49.22 (9.43)
≥80	541(54.10)	37.43 (9.94)***	48.97 (10.2)
**Marital status**			
Married	403(40.30)	42.42 (10.29)	50.25 (9.08)
Widowed/Divorced/Single/Separated	597(59.70)	38.23 (10.02)***	48.3 (10.26)**
**Chronic disease**			
Yes	928 (92.80)	38.95 (9.93)	48.80 (9.88)
No	72 (0.72)	52.40 (6.61)***	52.79 (8.70)***
**Hypertension**			
Yes	516 (51.60)	39.01 (9.89)	48.91 (9.50)
No	484 (48.40)	40.78 (10.67)**	49.25 (10.17)
**Diabetes**			
Yes	805 (80.50)	36.99 (9.58)	48.71 (9.99)
No	195 (19.50)	40.63 (10.39)***	49.17 (9.82)
**Heart problem**			
Yes	754 (75.40)	37.38 (9.89)	47.94 (10.03)
No	246 (24.60)	40.75 (10.34)***	49.46 (9.76)*
**Osteoarthritis**			
Yes	438 (43.80)	36.88 (9.21)	48.25 (10.09)
No	562 (56.20)	43.82 (10.39)***	50.16 (9.42)**
**Eye problem**			
Yes	596 (59.60)	37.38 (9.75)	47.87 (10.25)
No	404 (40.40)	41.64 (10.37)***	49.91 (9.49)**
**Ear problem**			
Yes	857 (85.70)	37.02 (9.93)	48.4 (9.36)
No	143 (14.30)	40.4 (10.32)***	49.2 (9.93)

* *P*≤0.05,

** *P*≤0.01,

*** *P*≤0.001

SF-12v2: Short Form-12 Health Survey Version 2; MCS-12: Mental Component Summary; PCS-12: Physical Component Summary.

The self-report questions about chronic diseases and health problems were asked by trained investigators. The participants were asked “Has a doctor ever told you that you had chronic diseases (for example, hypertension, diabetes, heart problem, osteoarthritis, eye problem, ear problem or others)?” Among the above questions, eye problems included age-related maculopathy, glaucoma, cataract, etc., while ear problems included otitis media, difficulty hearing, etc

## Discussion

Our study reported the reliability (internal consistency) and validity (convergent validity, discriminant validity, factorial validity, known-groups validity) of the Chinese version of SF-12v2, a widely used generic HRQoL instrument, among the elderly population in Guangzhou. The results provided strong evidence that SF-12v2 is a reliable, valid instrument for measuring and monitoring HRQoL in this population.

As PCS-12 and MCS-12 scores were calculated by norm-based scoring algorithms, our results constitute a reference for cross-cultural comparisons in the HRQoL domain. In addition, the data were 100% complete in valid questionnaires, as face-to-face questionnaire administration allowed data quality to be checked onsite; however, the findings might not hold with a self-administered questionnaire, an approach shown to lead to high incompletion (20).

Internal consistency reliability was >0.7 for the two component summary domains and the eight subscales, indicating that SF-12v2 is reliable for older adults in Guangzhou coming from different aged care models. This is higher than the reliability for the general population of Hong Kong (6) and comparable with that for older adults in southern Sweden (17). For both PCS and MCS, no floor or ceiling effects were observed, indicating that these summary scores sensitively measured variation in older adults’ health status.

SF-12v2 had good convergent and discriminant validity. Most items were strongly correlated with their represented component: the correlation of PF, RP, BP, and GH items with PCS was stronger than with MCS, and vice versa for RE, VT, and MH items. However, the correlation of SF item with PCS was unexpectedly a bit stronger than with MCS. Similar results were found in a cross-sectional postal survey of 8500 older adults in southern Sweden (17). For older adults, physical health might be an important factor in whether to participate in social activities (with friends or family members, at home or out) (21).

Even those who used to actively participate in social activities or were still very interested in doing so often found it difficult owing to poor physical health. Therefore, social functioning may be more highly related to the physical health component than the mental health component in the elderly population. What is more, items of RP and GH also had moderate correlations with MCS, items of SF had a relatively high correlation with MCS, and items of VT had a relatively high correlation with PCS. The reason might be related to the socio-demographic characteristics of older adults in China: the participants were generally not well educated, and varied greatly in income (22). Further adjustment to the instrument may thus be needed to suit Chinese culture better. Furthermore, older adults’ comprehension and memory ability generally deteriorate with age. Thus, their understanding of abstract concepts such as “vitality,” “general health,” and “social activity” might also vary. All the above factors might affect the subjective judgments of the participants. In agreement with the original SF-36, items of GH, SF, and VT subdomains correlated highly with both MCS and PCS (11, 23). RP items correlated moderately with MCS, which may be explained by noting that among Chinese older adults, RP items are understood to some extent as indications of mental as well as physical health. Similarly, items of RP, GH, VT, and SF were reported to correlate with both PCS and MCS (correlations≥0.4) in the Greek general population (7). However, in that study, VT items correlated more highly with PCS and SF items with MCS, a bit different from this study.

Principal components analysis with varimax rotation supported SF-12v2 with a two-factor structure, the original conceptual model of SF-12v2. All item–factor loadings were confirmed except for SF items, which loaded more highly on PCS than on MCS, and VT items, which tended to cross-load on both summary components. Similar to a general-population study in the city of Chengdu, two factors (physical and mental health) were extracted in exploratory factor analysis (24). What is more, a two-dimensional item structure was also found in southern Sweden among older adults with Parkinson’s disease, though that study identified a three-dimensional item structure among general older adults and older adults with stroke (17). A Korean general population study also reported a three-factor structure in exploratory factor analyses (8). Moreover, here, confirmatory factor analysis indicated that the theoretical two-factor structure fitted the data from older adults in Guangzhou very well. Many studies show similar results in general populations (9, 10) and people with specific diseases (11, 12); what is more, a study among Chinese older adults in central Shanghai also found that a two-factor structure fit the data well (25).

SF-12v2 summary scores differentiated subgroups of older adults through known-groups validity, by age, marital status, and self-reported health problems, showing evidence of construct validity. Consistent with previous studies (10), our findings also showed that older adults with chronic disease status had poorer SF-12v2 summary scores than those without chronic disease; similarly, older adults who reported hypertension, diabetes, heart problems, osteoarthritis, eye problems, or ear problems had significantly lower mean PCS-12 and MCS-12 scores than those without. These results are consistent with results of studies in Greece (7) and Italy (26).

There were a few limitations to this study. First, given the cross-sectional design, we could not provide evidence for test–retest reliability or longitudinal construct validity of SF-12v2. Second, participants were likely healthier than older adults in general, as they were able to self-report. Third, recall bias might be present, due to the self-report method. The generalizability of study results may be limited by these factors, and further studies on the psychometric properties of the Chinese SF-12v2 among older adults are needed. Despite these limitations, SF-12v2 demonstrated validity and reliability among an elderly population in Guangzhou, a typical big city in China.

## Conclusion

SF-12v2 works equally well in older adults under an institutional care model and under a community-based home care model. The psychometric performance of SF-12v2 was satisfactory to indicate the health of these Chinese older adults, scientifically justifying the use of this health measurement tool with them. This will simplify the use of health indicators for older adults in clinical studies, especially large-scale ones. As the population ages further, healthcare expenditures will increase. Selecting suitable HRQoL measurement and monitoring tools for older adults will help manage their health in advance or predict major issues, promoting healthy, active aging.

## Ethical considerations

Ethical issues (Including plagiarism, informed consent, misconduct, data fabrication and/or falsification, double publication and/or submission, redundancy, etc.) have been completely observed by the authors.
